# Contribution of cellulosic fibre filter on atmosphere moisture content in laser powder bed fusion additive manufacturing

**DOI:** 10.1038/s41598-019-50238-5

**Published:** 2019-09-24

**Authors:** A. Das, J. A. Muñiz-Lerma, E. R. L. Espiritu, A. Nommeots-Nomm, K. Waters, M. Brochu

**Affiliations:** Department of Mining and Materials Engineering, McGill University, 3610 University Street, Wong Building, Montreal, QC H3A 0C5 Canada

**Keywords:** Engineering, Engineering, Engineering, Soft materials, Soft materials

## Abstract

Cellulosic materials are commonly used to manufacture the particulate filters used in laser powder bed fusion (LPBF) additive manufacturing (AM) equipment. An experimental approach has been used to calculate the moisture quantity and kinetics of sorption in a cellulosic filter at varying relative humidity (RH) levels. A prediction of the amount of moisture which can be theoretically held within a filter during storage before its use has been obtained. Subsequently, the quantity and the rate of moisture desorption which can be transferred into the build chamber during LPBF is presented. This work highlights the importance of filter storage and conditioning prior to use in additive manufacturing processing.

## Introduction

Powder bed fusion additive manufacturing is a layer-by-layer manufacturing technique that allows the fabrication of complex parts with enhanced functionality^1^. The successful adoption of AM technology has been fostered by improvements in powder feedstock quality and process optimization^2–5^. A key factor to be controlled in AM processing is the oxygen concentration within the build environment^6,7^. During laser powder bed fusion (LPBF) processing, oxygen concentration has been found to influence the composition of various alloys such as Ti-6Al-4V^8,9^, Al-12 Si^10^, IN 718^11^, 316 L SS^12^ and 17 PH SS^12,13^. In some metallic systems like Ti-6Al-4V, the oxygen content even dictates the grade of the material (grade 23 vs grade 5), and ultimately the mechanical properties of the part^14,15^.

The oxygen concentration variation in the built part can be affected by a variety of factors. It has been hypothesized that oxygen contributions can come from adsorbed moisture present on the powder surface and in the filter, as well as inert gas quality contamination. Within this list, moisture, which is not always monitored, must be controlled since its reaction with the melt pool and the surrounding high-temperature region can augment the oxygen contamination in the produced parts.

Metallic powders have been found to be one of the predominant sources which contribute moisture to the AM environment^3^. Work has shown that moisture can be adsorbed onto the powder surface through water-metal interactions and water-water hydrogen bonding^16^. The quantity of moisture adsorbed is alloy dependent, with surface roughness, the presence of oxide and surface impurity concentration all independently contributing^17^. Amongst other moisture sources, commercial ‘high purity’ argon gas, for example, has 3 ppm (by volume) of moisture contained within it^18^. This highlights the fact that even in a perfectly sealed AM unit, with dried AM powders, inherent moisture will still be present.

The filter is critical in the operation of the AM system, as it removes the airborne particles produced during the building process. An understanding of its behaviour within the build environment and its possible contribution of moisture to the machine atmosphere is required. Filters for AM are commonly fabricated using pleated cellulose paper^19,20^ composed of wood fibres^21^, which are formed from micro-fibrils of chainlike cellulose aggregates^22^. Cellulose has a semi-crystalline structure; it possesses an amorphous region with a high amount of available hydroxyl groups that have the potential to interact with moisture through hydrogen bonding^22,23^. In the presence of a certain amount of water vapour, a dynamic concurrent phenomenon of adsorption and desorption of the water molecules from the fibres continues until an equilibrium moisture content (EMC) is reached^24^. To date, no published literature is available assessing the contribution of moisture from filters in the LPBF AM environment.

Upon storage in an uncontrolled environment, filters can adsorb moisture from the atmosphere. LPBF AM operation is conducted in a controlled environment involving inert gases. During printer operation, the adsorbed moisture in the filter can potentially desorb leading to a reaction with the melt pool. This reaction can lead to an increase in oxygen content in the part and powders^25^.

The presence of moisture in LPBF environment can lead to other issues. Research has shown that moisture present during processing has been linked with defects such as surface micropores and variations in density in parts produced by LPBF AM^10,26^. For this reason, Bourell *et al*.^27^ recommended a thermal degassing pre-treatment prior to the laser processing of titanium powders to eliminate its effect; this highlighted the importance of moisture control in AM processing. For LPBF AM of titanium systems, it has been recommended to keep the cumulative oxygen and moisture content in the build chamber below one ppm^28^.

The aim of the present work was to study the moisture sorption and desorption from an AM filter in order to quantify the possible moisture contribution during the printing process. Dynamic vapor sorption (DVS) analysis has been used to evaluate the sorption and desorption capability of an industrial AM filter to understand its behaviour during filter storage. Additionally, real-time moisture monitoring during chamber atmosphere preparation of an industrial AM unit was carried out. This contributes to filling the current knowledge gap present by identifying the filter as a source of moisture and assessing its contribution within the build chamber during processing in LPBF AM.

## Materials and Methods

The contribution of cellulosic fibre filter on the atmospheric moisture content in LPBF additive manufacturing was studied using E 1900L cellulosic filters sourced from Hengst SE, Germany, purchased from Renishaw Canada, Ltd. The filter was tubular in design, with a rubber top and base to hold concertinaed filter paper around the periphery, the filter dimensions were; 401 mm high, with an outer diameter of 195 mm and an internal hollow diameter of 118 mm.

Filters were characterised with a variable pressure Scanning Electron Microscope (SEM) using a Hitachi SU3500 at a gas pressure of 70 Pa and a beam energy of 10Kv. The specific surface area of the filter was measured via the Brunauer–Emmett–Teller (BET) method using a Micromeritics TriStar 3000 (Micromeritics Instrument Corporation, USA) gas sorption system.

The filters were received packaged in cardboard boxes with no internal wrapping. Throughout the study, the environmental RH measurements were carried out using a Traceable Memory Hygrometer (Fisher Scientific, USA). To determine the storage RH conditions of the filters, a set of measurements were taken inside the laboratory and from the inside of the cardboard storage container by inserting the hygrometer probe through 3 different filter storage containers.

The quantification of the moisture sorption and desorption capacity of the studied filter was carried out using the gravimetric dynamic vapor sorption (DVS) technique (DVS Intrinsic Plus, from Surface Measurement Systems, London, UK). The DVS Intrinsic apparatus measures mass change (±0.1 µg) under controlled temperature and humidity. Prior to testing, circular samples were punched from the filter using an APSCO 330A paper hole punch with a diameter of 6.35 mm (0.25 inch) and dried in an air atmosphere at 80 °C for 1 hr. Dried samples were loaded into an aluminium pan and placed into a chamber at a controlled temperature of 25 °C and allowed to reach equilibrium, i.e., until the change in mass as a function of time was less than 0.002 % per minute. The sorption tests started by soaking the samples for 6 h in a flow of dry air to allow the chamber to reach 0 % RH. After 6 hrs of gas drying, the RH in the DVS chamber was increased in steps of 10 % RH until 80 % RH was reached. Each RH step was held until equilibrium. Once 80 % RH was reached, the desorption test started by ramping down the RH from 80 % to 0 % in steps of 10 % mirroring the ramp up. To verify the DVS data for the moisture sorption capacity of the filters, three packaged filters stored under identical conditions were unpacked and consequently oven dried at 90 °C for 5 hrs. The RH of storage within the laboratory for this case varied between 31 and 35 % over a period of 48 hrs.

Once the adsorption and desorption capacity were measured via DVS, *in situ* measurements of RH during the chamber atmosphere preparation of a Renishaw AM250 with no powder were conducted. The RH variation as a function of time was recorded using a Fischer brand probe hygrometer installed in the build chamber. The LPBF machine used in the present study has the option to select between two different chamber atmosphere preparation methods. The first method denominated as *“with evacuation”*, consists of an evacuation step followed by a purging process. The evacuation step consists on extracting the gases present in the build chamber using a mechanical vacuum pump until a vacuum pressure of 40 mBar is reached. The purging process is carried out by filling the build chamber with Ar gas followed by the simultaneous injection and venting of Ar until the target oxygen concentration in the chamber was reached. The second chamber atmosphere preparation method denoted as *“without evacuation”*, lacks an evacuation step and only presents a purging process that is carried out until the target oxygen concentration was reached. In both methods, gas recirculation through the filter was periodically induced during the purging process. However, when the target concentration of oxygen was reached, gas recirculation was continuous until the printing process started.

In the present study, both methods, “*with evacuation”* and *“without evacuation”*, were used with a gas recirculation time of 5 min after 1000 ppm of oxygen concentration was reached. Three filter conditions were tested by both chamber atmosphere preparation methods for a total of six testing conditions. In order to identify a baseline value of RH, both chamber atmosphere preparation methods were conducted without a filter. The following two tests were conducted using filters dried at 90 °C for 5 hr. Finally, the last two tests were carried out using as-received filters stored under laboratory environment and denominated as ‘non-dried filter’.

## Results and Discussions

### Filter characterisation

**Figure 1:**
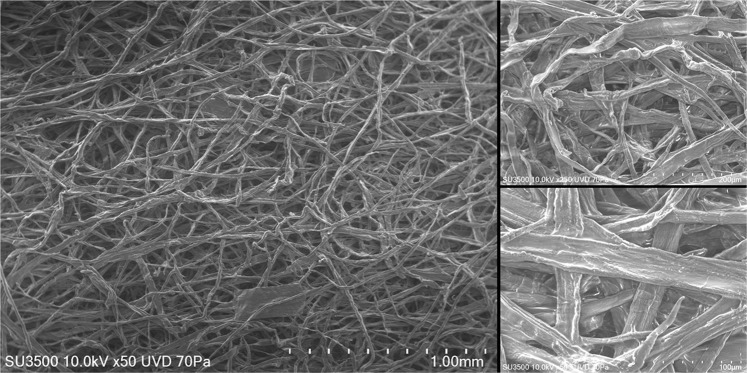
SEM images of the as-received filter surface at 50, 250 and 500x magnification.

Figure 1 depicts a representative surface of the as-received filters. As shown, the filter is made up of individual fibres of ~20 μm in diameter forming a spaghetti like structure; their surface roughness was typical for cellulosic materials. Surface area was measured via BET and was found to be 0.2491 m^2^/g.

### Water vapor sorption and desorption characteristics

**Figure 2:**
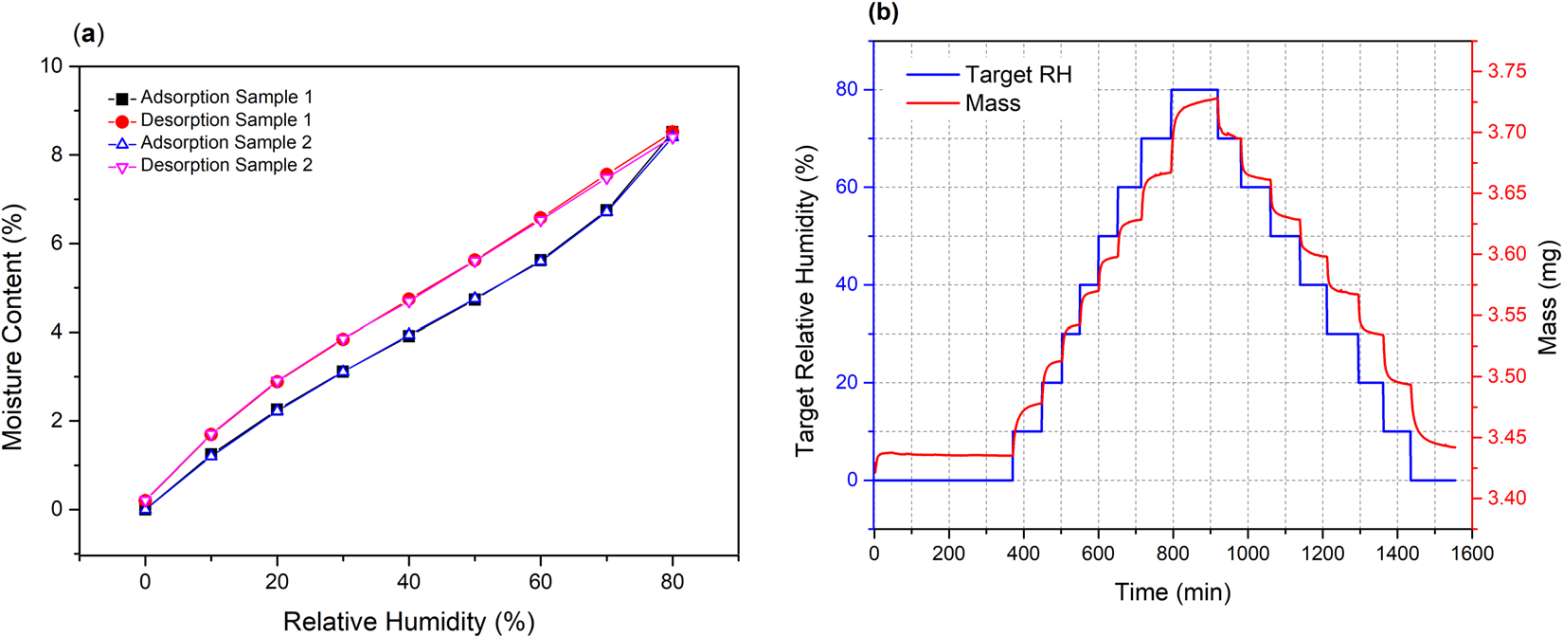
(**a**) DVS sorption/desorption isotherm loop for 2 samples, and (**b**) change in mass of sample 1 in response to the set RH values of the DVS sorption/desorption isotherm loop.

The DVS was utilised to determine the EMC of the filter as a function of the environmental RH. Figure 2(a) shows representative DVS sorption and desorption isotherm loops for two samples of filter paper. This data indicates that the sorption and desorption is a reversible process dependent on the subjected external RH. A hysteresis was observed as the EMC values are slightly higher upon desorption to the same RH level. As demonstrated, the reproducibility of the hysteresis was consistent. Figure 2(b) depicts the mass gain (during sorption) or mass loss (during desorption) for Sample 1. The blue coloured steps indicate the machine command (or target) stepwise increase/decrease in RH within the DVS chamber. Corresponding to this RH change, the red coloured steps indicates the change in mass of the specimen. It can be observed that this mass rise (on sorption) and mass loss (on desorption) was initially fast and stabilized by gradually slowing down to reach the EMC value. For each of these steps in the DVS, stabilization to EMC required between 1–2 hrs.

In order to explain the observed fast and slow mass change, a Parallel Exponential Kinetics (PEK) model was used to fit the data. In literature, this model was successfully used to model the DVS behaviour of various natural fibres including jute^21,22^, coir^22^, flax fibre^21,22^, and hemp^21^. The PEK is based on a double exponential constituted of a fast and a slow step of moisture adsorption/desorption. Several mechanisms of moisture adsorption between the slow and fast processes have been proposed^29–32^, but a coherent understanding of their physical meanings remains to be firmly established^21,24^. Hill *et al*.^24,33^ postulate that the fast process in the PEK model represents adsorption or desorption of water molecules on readily available OH (hydroxyl) sites. The slow process in the PEK model has been associated with two possibilities, either to a gradual diffusion of moisture at less accessible sites within the fibre, or to swelling or shrinking of the fibre as sorption proceeds towards equilibrium^24^.

Desorption within the machine may include a fast and a slow process constituting the exponential decay in the RH. This suggests a PEK type of model for data fitting. Interpretation of the PEK model fitting is complicated as the moisture allocation between the fast and slow processes is yet to be understood. For a better understanding of the physical process, a simple single exponential decay can be used because it is deemed sufficient to explain the initial fast and gradual slowdown phenomena as given in Eq. (1),

1 $$ \% \,{\rm{RH}}= \% \,{{\rm{RH}}}_{{\rm{Stabilised}}}+{{\rm{C}}}^{\ast }\exp (-{\rm{t}}/{{\rm{t}}}_{{\rm{1}}})$$

% RH refers to the RH within the chamber at time t, the equilibrium amount of moisture content within the chamber correspond to the % RH_Stabilised_ value, C is a constant indicating the difference between the initial and stabilised RH and t_1_ is the characteristic time (inverse of rate constant) which provides an indication of the length of time required for the RH content to stabilise.

#### Relating external storage RH to the packaged filter RH

The mean RH measured in the laboratory atmosphere and the filter storage box was 57.55 % (s = 0.45 %) and 56.11 % (s = 1.04 %) respectively. As indicated, a minor difference was observed. The similarity between the RH content outside and inside the storage box has been attributed to the permeability of the cardboard storage box^34^. Moreover, the cardboard boxes had openings due to folding at the corners that can facilitate air exchange with the local environment. Therefore, in the case of stored filters in cardboard boxes, the internal RH value can be assumed to be approximately equal to the RH value outside of the box. In that context, the EMC within the filter is dependent upon the local RH in which the sample is stored, therefore, changes in geographical location and seasons will have significant effects on the moisture content present.

#### Quantity of moisture held in a filter during storage

In reality, filters might be stored for days or weeks before being used in a printer. During storage, they can accumulate moisture from the surroundings. DVS results have shown that it took 1–2 hrs for the filter paper to reach EMC values for any RH increment/decrement step (Fig. 2(b)). Therefore, it can be assumed that if the filters are stored for several days, they will reach their EMC values if the ambient RH is constant. For complete oven drying at 90 °C, the duration of five hours was found to be sufficient as the filter weight remained constant beyond 5 hrs. Upon such drying conditions, three filters from long-term storage (31–35 % RH variation in past 48 hrs) exhibited a mean moisture loss of 3.85 % with a low standard deviation of 0.28 % among the set. This shows each filter contained similar amounts of moisture compared to other filters stored under the same conditions. This moisture content is within or close to the EMC values corresponding to 31–35 % RH in the sorption isotherm loop obtained by the DVS (Fig. 2(a)). Thus, during storage, the filter either sorbs or desorbs moisture to reach the EMC value corresponding to the external RH value. Therefore, the EMC values obtained using the DVS experiment can be multiplied by the dry weight of the filter to obtain the mass of stored moisture (scale up).

**Figure 3:**
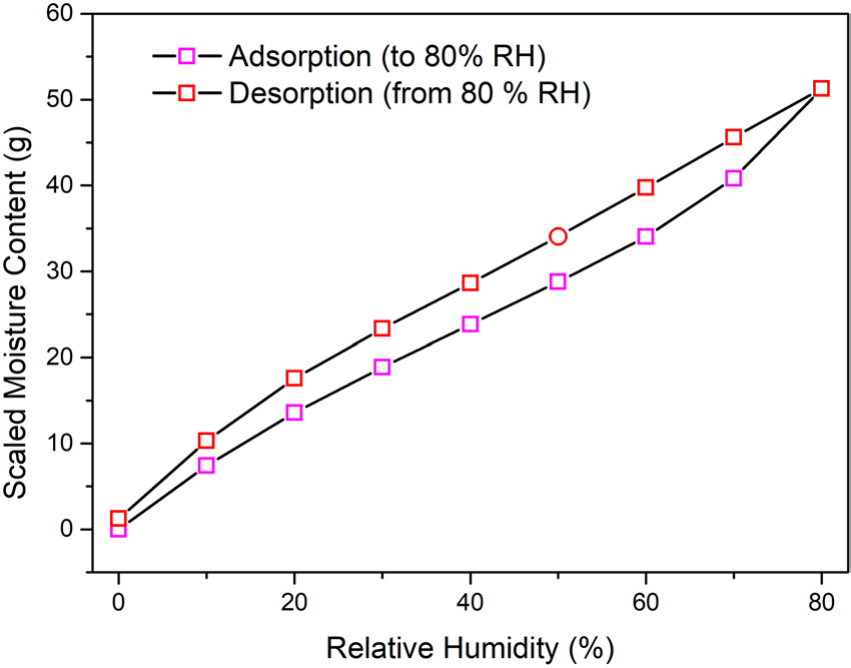
Scaled value of moisture content within a filter (mean dry weight 606.35 g) during sorption and desorption up to 80 % RH.

Figure 3 provides this equilibrium scaled value of the mass of water stored within an actual filter at various RH values. To construct Fig. 3, the mean dry weight of 606.35 g obtained by drying three filters was used for multiplication with the EMC values from the DVS sorption isotherm loop (Fig. 2(a)). As demonstrated, the EMC of the filter can vary significantly with storage humidity; i.e. the filter adsorbs a minimum of (values along adsorption line) 0–51.28 g of moisture corresponding to storage at 0–80 % RH.

In the presence of a low RH environment, the adsorbed water on a filter can be desorbed. The chamber atmosphere preparation cycle in a LPBF AM system, either by evacuating the present gas or by purging with an inert gas, creates a low RH environment, therefore facilitating moisture desorption from the filter. Thus, it is reasonable to think that during LPBF processing, the filter can act as an undesired source of moisture.

### Moisture desorption from filters during chamber atmosphere preparation

#### LPBF AM build chamber with no filter unit

**Figure 4:**
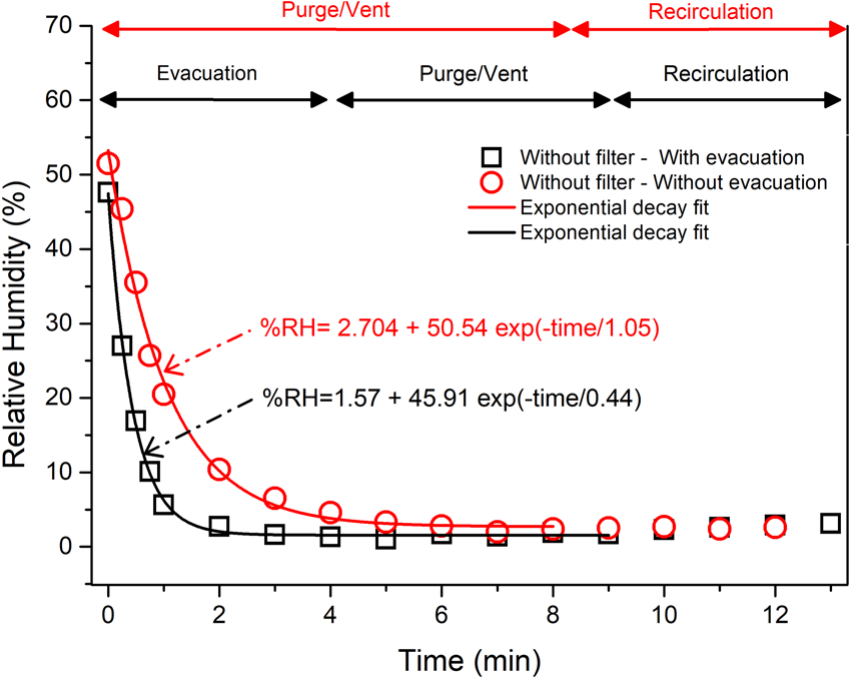
Variation of RH within the LPBF build chamber without a filter unit when subjected to a chamber preparation cycle without evacuation (red line) and with evacuation (black line).

Figure 4 shows the change in measured RH during chamber atmosphere preparation with and without evacuation within the printing chamber when no filter unit is present. The initial chamber atmosphere preparation with or without evacuation follows an exponential decay pattern as represented by Eq. (1).

As visualized in Fig. 4, the experiment with evacuation initially had a faster decay rate which was around twice as fast when compared to the absence of evacuation. Additionally, the RH also stabilised quicker reflected by the lower characteristic time t_1_ (0.44 min or 26.4 s compared to 1.05 min or 63 s). This shows that evacuation was more effective than purging gas and venting out moisture from the build chamber.

Upon recirculation, RH values for both experiments remained stable at around 1–3 %, which is within the tolerance limit (4 %) of the measuring hygrometer^35^.

#### LPBF AM build chamber with a dried filter unit

**Figure 5:**
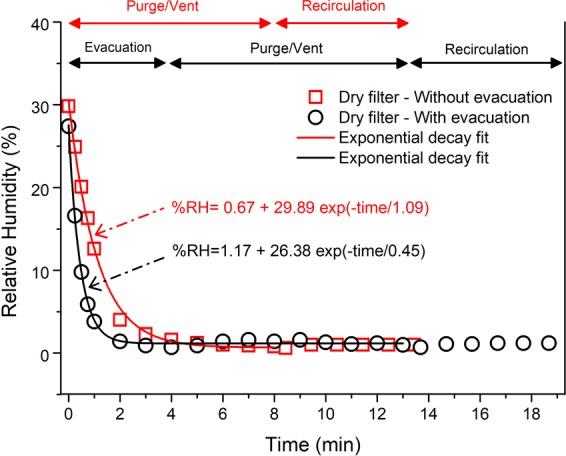
Variation of RH within the LPBF build chamber with a dried filter unit when subjected to a chamber preparation cycle without evacuation (red line) and with evacuation (black line).

Figure 5 shows the relationship between RH and purge cycles conducted on an oven dried filter. During the initial chamber atmosphere preparation with a dry filter, irrespective of the presence of evacuation, an exponential decay in RH was observed with similar characteristic times as the experiment without the filter. Therefore, the removal of the moisture trapped in the chamber during either of the chamber atmosphere preparation was similar to the tests conducted with no filter unit (Fig. 4). After 5 mins of recirculation, for both cases, the filters had low stabilised RH values which were preserved in contrast to those observed in the case of the experiments with the non-dry filter.

#### LPBF AM build chamber with a non-dry filter unit

**Figure 6:**
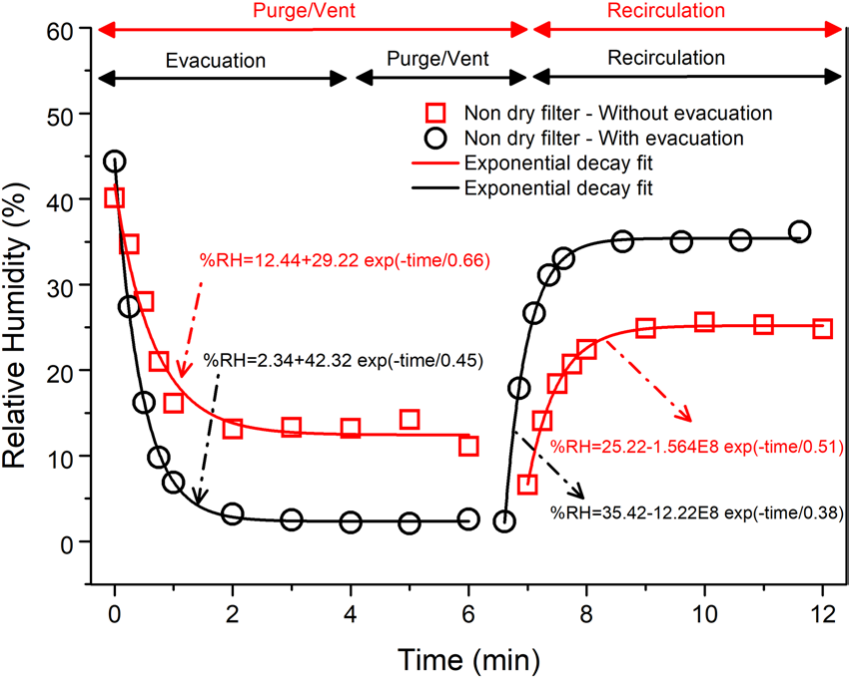
Variation of RH within the LPBF build chamber with a non-dry filter unit when subjected to a chamber preparation cycle without evacuation (red line) and with evacuation (black line).

Figure 6 shows the change in RH with a single printer chamber atmosphere preparation cycle with and without an evacuation when a non-dry filter unit was used. In the initial stage, for both cases, the RH decays exponentially and can be described by Eq. (1). The characteristic times for the experiment with evacuation was similar to the test without a filter and with a dried filter. This indicates a maximum value of chamber atmosphere moisture removal rate during the evacuation.

The experiment without evacuation had two major purging and venting cycles. Therefore, the decrease in RH was in two steps, the initial first step and the second step beginning after 6 min. The observed initial RH drop was smaller when compared to the experiments without a filter and a dried filter. Thus, a shorter characteristic time was observed compared to the corresponding experiment without the filter and with a dried filter. Additionally, in the entire initial stage, it had a higher stabilized RH content compared to every other experiment; this points towards a supply of moisture from the non-dry filter during gas purging.

Upon recirculation of argon gas, the RH values in the chamber rose (Fig. 6) and thereafter stabilised for both cases following an exponential decay pattern (Eq. (1)). The observed rise in RH occurs due to desorption of water molecules from the filter interacting with the dry gas. The initial rapid gain of RH can be explained by a high rate of moisture release by the readily available desorption sites on the filter paper surface. The higher stabilised RH and shorter characteristic time for the experiment with evacuation indicate higher moisture content. The higher moisture content also readily translates into the presence of a higher number of available desorption sites which contributes to a faster desorption rate on recirculation. With time, gradually the contribution of desorption from the readily available desorption sites is reduced and desorption occurs from the lesser accessible sites. Thereafter, as the gas recirculated a steady equilibrium RH value was established within the machine.

For both experiments, the stabilised RH value after recirculation was lower compared to the initial RH, reflecting moisture loss from the filter. Table 1 predicts an upper bound of the amount of moisture which could be reduced, utilizing the adsorption data from the scaled moisture content given in Fig. 3. The RH value within the laboratory was the same as the initial RH measured in the experiment, as the LPBF system was open to the laboratory atmosphere prior to starting the experiment. In both experiments, the two filters have close initial RH values. Thus, they contained similar quantities (mass %) of moisture within them. Without evacuation or solely by purging/venting, this reduction of moisture content was greater than the experiment with the evacuation. The greater reduction can be attributed to the flowing inert gas within the system in case of purging which can interact with the filter causing faster desorption than in case of evacuation where gas flow was absent.

**Table 1:** Prediction of moisture loss during initial chamber atmosphere preparation for a non-dry filter.

Experimental Process	RH value Initial (%)	RH value Stabilised (%)	Maximum Predicted Moisture Loss (g)
With evacuation	44.66	36.43	3.84
Without evacuation	41.64	25.27	7.68

## Conclusion

This study utilises DVS and LPBF machine experiments to gain an understanding of how the filter cartridge can contribute to the moisture content in a LPBF AM system. Notable conclusions which can be drawn are;

1. Filters used in LPBF machines can adsorb moisture during storage unless sealed properly from the atmosphere or stored in zero RH environments. The amount of moisture adsorbed depends on the RH in which it was stored. The predicted amount of adsorbed moisture during storage ranged from 0–51.28 g corresponding to 0–80 % environmental RH for a Hengst E1900 L air filter as shown in this study.
2. The filter loses some of its adsorbed moisture during both the chamber atmosphere preparation processes of evacuation and inert gas purging. The amount and rate of moisture loss are dependent upon the initial moisture stored in the filter and the parameters of the chamber atmosphere preparation processes. In this study, greater desorption was observed in the case of inert gas purging compared to chamber evacuation from a non-dry filter.
3. Moisture in the LPBF chamber during processing can arise from the non-dry or a non-preconditioned filter unit. In the case of gas recirculation, the cyclic flow of inert gas established an RH equilibrium in the LPBF system.
4. Preconditioning the filter by drying for 5 hrs at 90 °C, successfully removed the entire moisture from the filter. An alternative strategy could be to store the filter in a place with low RH.

Although the exact contribution of the filter to the oxygen content will depend on a number of variables related to the specific LPBF equipment and the laser processing parameters, this study highlights the role of the filter in introducing moisture in the LPBF AM system. In absence of appropriate countermeasures, this can lead to an undesired increase of oxygen content in the expensive powders and parts.

## Data Availability

The experimental data presented in this paper is available for sharing upon request.
